# Modelling risk-adjusted variation in length of stay among Australian and New Zealand ICUs

**DOI:** 10.1371/journal.pone.0176570

**Published:** 2017-05-02

**Authors:** Lahn D. Straney, Andrew A. Udy, Aidan Burrell, Christoph Bergmeir, Sue Huckson, D. James Cooper, David V. Pilcher

**Affiliations:** 1 School of Public Health and Preventive Medicine, Monash University, Melbourne, Victoria, Australia; 2 Department of Intensive Care and Hyperbaric Medicine, The Alfred Hospital Melbourne, Victoria, Australia; 3 Faculty of Information Technology, Monash University, Melbourne, Victoria, Australia; 4 Australian and New Zealand Intensive Care Society, Centre for Outcome and Resource Evaluation, Melbourne, Victoria, Australia; University of Florida, UNITED STATES

## Abstract

**Purpose:**

Comparisons between institutions of intensive care unit (ICU) length of stay (LOS) are significantly confounded by individual patient characteristics, and currently there is a paucity of methods available to calculate risk-adjusted metrics.

**Methods:**

We extracted de-identified data from the Australian and New Zealand Intensive Care Society (ANZICS) Adult Patient Database for admissions between January 1 2011 and December 31 2015. We used a mixed-effects log-normal regression model to predict LOS using patient and admission characteristics. We calculated a risk-adjusted LOS ratio (RALOSR) by dividing the geometric mean observed LOS by the exponent of the expected Ln-LOS for each site and year. The RALOSR is scaled such that values <1 indicate a LOS shorter than expected, while values >1 indicate a LOS longer than expected. Secondary mixed effects regression modelling was used to assess the stability of the estimate in units over time.

**Results:**

During the study there were a total of 662,525 admissions to 168 units (median annual admissions = 767, IQR:426–1121). The mean observed LOS was 3.21 days (median = 1.79 IQR = 0.92–3.52) over the entire period, and declined on average 1.97 hours per year (95%CI:1.76–2.18) from 2011 to 2015. The RALOSR varied considerably between units, ranging from 0.35 to 2.34 indicating large differences after accounting for case-mix.

**Conclusions:**

There are large disparities in risk-adjusted LOS among Australian and New Zealand ICUs which may reflect differences in resource utilization.

## Introduction

Healthcare providers routinely evaluate their quality of care using patient-centered outcomes such as complications, readmission rates, and mortality.[1, 2] Comparisons of mortality rates using risk-adjustment models is a common approach for evaluating intensive care unit (ICU) performance,[3, 4] however it is of interest to consider variation in outcomes within the context of resource use.[5, 6] That is, are some units achieving positive patient outcomes with low resource use while others are using considerably more?

The relevance of length of stay (LOS) as a measure of performance in the ICU is two-fold. First, it relates to the efficiency of the intensive care process, and thus cost within the ICU and the institution overall. The majority of the cost associated with ICU patients is related to bedside staffing required for patient care, which is a direct function of patient LOS. Second, it may serve as an indirect marker of the quality of care; more effective therapy results in more rapid recovery, while complications and errors potentially result in extended LOS.

For objective comparison of resource use among institutions, LOS requires adjustment for patient case mix.[7, 8] Prediction models can be employed to account for much of the variability in patient case mix, as well as other admission factors, such as source of admission. Subsequently these models can be used to obtain reasonable estimates of the effect of the institution on patient LOS, after controlling for patient-level factors. Quantification of institutional variation of patient LOS among ICUs has important implications for health care management. In particular, it may serve as a tool for recognition of potentially inefficient practice and sub-optimal quality of patient care.[9, 10]

The primary aim of this study was to develop a model for predicting ICU LOS, utilizing individual patient-level characteristics, thereby providing a risk-adjusted metric to allow comparison of length of stay between ICUs in Australia and New Zealand (ANZ). Our secondary aim was to describe the validation framework for evaluating model performance, in the context of assessing unit variability.

## Methods

### Study design and setting

The Adult Patient Database (APD) is one of four clinical quality registries run by the Australian and New Zealand Intensive Care Society (ANZICS) Centre for Outcome and Resource Evaluation (CORE). The APD collects patient-level data for all admissions to adult ICUs throughout ANZ. Commenced in 1993, the APD now includes over 1,600,000 patient episodes.

Variables include patient sex, age, primary admission diagnosis (assigned using the ANZICS modification of the APACHE-III diagnostic codes),[11, 12] physiological and biochemical measures from the first 24 hours of ICU admission, goals of treatment, and requirement for mechanical ventilation. Calculated variables such as the Australian and New Zealand Risk of Death (ANZROD) model, were also analyzed.[3] ANZROD is the primary risk adjustment method for comparing mortality outcomes within Australia and New Zealand and has been shown to provide better risk adjustment than the APACHE III scoring system.[4]

Institutional approval to undertake the study was provided by The Alfred Hospital Human Research Ethics Committee (Melbourne, Australia) with a waiver of individual patient informed consent (Project No. 259/15).

### Inclusion/Exclusion criteria

We extracted de-identified data from the ANZICS APD for patient admissions between January 1 2011 and December 31 2015. We excluded patients with a LOS greater than 180 days (n = 258, 0.04%). We were concerned that an unknown number of these patients’ LOS may be due to data error and were unable to assess their validity. We excluded patients that were missing ANZROD scores or aged <18 years at the time of admission.

### Statistical analysis

#### Model building

Given the typical right skew of LOS, we used log transformed LOS as our primary outcome (ln-LOS). Our prediction model was built using a multi-stage process. First, to assess the inclusion of diagnosis in the model, we separately tested each diagnosis in a log-linear regression model with the deciles of ANZROD as a categorical control variable. Z-scores for each diagnosis were calculated by dividing the coefficient by the standard error. All diagnoses were ranked by the absolute value of their Z-score. To balance predictive power with a desire to be parsimonious and avoid over-fitting, the top 50 conditions were considered for inclusion in the model (these accounted for 62.1% of all admissions). Second, all diagnoses were entered into a saturated model including other patient and admission characteristics and the resulting coefficients were used to classify the diagnoses into categories. We grouped diagnoses into four long stay groups (β = >0.5, >0.3, >0.2 and >0.1) and three short stay groups (β = <-0.3, <-0.2, <-0.1). Diagnoses with coefficients -0.1 to 0.1, as well as those conditions not considered for inclusion were used as the reference category. Third, a mixed-effects linear regression was used to predict an individual’s Ln-LOS using diagnostic category with other patient and admission characteristics. A full description of coding for individual variables is available from the APD data dictionary.[11]

A p value of <0.05 was considered statistically significant. Non-significant variables were removed in a backward stepwise manner. Given large datasets can lead to spurious associations, we then reran the model on a 20% random sample and again removed non-significant variables in a backward stepwise manner. The final model coefficients and standard error estimates were calculated by bootstrapping with 200 repetitions using the entire dataset. The random effect was used to account for clustering within ICUs.

#### Calculating the risk-adjusted LOS ratio (RALOSR)

The expected Ln-LOS was defined only by the fixed portion of the model. The mean observed and expected Ln-LOS were calculated for each site and year. The exponent of the mean observed Ln-LOS is equivalent to the geometric mean of the LOS. We calculated a risk-adjusted LOS ratio (RALOSR) by dividing the geometric mean of observed LOS for each ICU by the exponent of the predicted Ln-LOS. The RALOSR is scaled such that a value less than one indicates a geometric mean LOS that is shorter than predicted, while a value greater than one indicates a geometric mean which is longer than predicted.

#### Validation framework

We assessed the performance of the model at the individual level using the concordance correlation coefficient and the coefficient of determination (R^2^) to assess agreement between the observed Ln-LOS and the predicted value [12]. Given the model is not intended to be applied at the individual patient level we used the same statistic to assess agreement between the mean observed and predicted Ln-LOS at the site level, and across patient and hospital subgroups. We use the term predicted value to refer to the estimate derived from the fixed and random effect, and the term expected value to refer to the estimate derived from the fixed effects only. One of the limitations of applying traditional validation techniques to a case-mix adjustment model is that they measure agreement between the observed and predicted to assess performance, even though we are expecting and interested in the error between the observed and predicted. Poor predictive performance at the individual and unit level may indicate a lack of adjustment for important confounders or be attributable to significant differences in discharge practices among units. [13] Thus poor model performance may not be indicative of poor utility of the model given its purpose is to highlight disparities in practice. For this reason, two novel approaches were employed to evaluate model performance in the context of our objectives.

First, as residuals in continuous models are typically correlated with outcome, the site RALOSR is likely to be correlated with the site’s geometric mean LOS. However, the RALOSR would not be useful if it simply reflected the mean LOS in each site. If we consider the extreme examples, sites with a very low mean LOS and with a very high mean LOS are likely to also have very low ratio and very high ratio respectively. Very high or low LOS is likely to reflect patient management practices, yet such sites are also likely to drive strong correlation between the RALOSR and observed LOS. We reasoned that risk-adjustment of LOS should mitigate the association between the ratio and mean LOS among the more ‘typical’ units. We defined ‘typical’ units as those with a mean LOS between the 25^th^ and 75^th^ percentiles. We measured the correlation between the RALOSR and mean LOS among all and these ‘typical’ units to assess adequacy of adjustment.

Second, in attributing differences in the ratio to differences in patient management and discharge practices, we reasoned that the RALOSR might change over time, but should not fluctuate from year to year. Meaning the measure, in a given unit, should not suggest very high resource use in one year, low resource use in the following year, and then very high resource use again in the following year. To assess this, we fitted a linear mixed effects model, with a fixed effect on year, a random intercept, and a random slope on year, thereby allowing separate linear trends and levels for each site. Fluctuating indices would result in poor correlation between the fitted value and the RALOSR. A reliable measure should have high correlation between the fitted values and the RALOSR.

#### Uncertainty intervals and sensitivity analyses

Small patient numbers may yield unreliable estimates of the RALOSR. Using those sites with >1000 admissions in a year, we assessed the number of admissions that needed to be observed before getting a result consistent with the estimate using all data. We randomly arranged admissions in each site in each year and calculated the RALOSR at the n^th^ admission. Specifically we took a subsample of size n from each site and calculated the RALOSR in each of these subsamples. We assessed the level of agreement with the final RALOSR (calculated using all admissions) with estimates calculated with n = 5, 10, 50, 100, 200, 300, 500 and 1000 admissions at varying tolerance levels (within 5%, 10%, 15%). We also examined the number of sites which would be misclassified as having significantly shorter or longer LoS based on interim estimate at n = 5, 10, 50, 100, 200, 300, 500 and 1000 admissions when compared to their final classification. A description of the method for calculating the uncertainty intervals is provided in the supplementary material.

## Results

### Demographics

There were a total of 662,525 admissions during the study period to 168 different ICUs. Characteristics of the admitted population are detailed in Table 1. The majority of patients were over 65, male, and were admitted post-surgery (Table 1). The median length of stay over the study period was 1.8 days (IQR: 0.9–3.5). The mean observed LOS was 3.21 days over the entire period, and declined on average 1.97 hours per year (95%CI:1.76–2.18). With the exception of hospital admission source, missing data was missing for less than 0.5% of admissions and these admissions were excluded from the analysis. An unknown hospital admission source was used as a discrete category in the model (Table 1).

**Table 1:** Characteristics of adult patients admitted to Australian and New Zealand ICUs 2011–2015.

Variable		Total^a^
**Total**	N	662,525 (100.0%)
**Length of Stay**	Mean (days)	3.21
	Geometric mean (days)	1.86
	Median (IQR) days	1.79 (0.92–3.52)
**Age**	Mean (SD) years	62.0 (17.5)
	Median (IQR) years	65.0 (51.0–75.3)
**Sex**	Male, % (N)	57.8% (382,852)
**Admission**	Elective, % (N)	43.2% (285,172)
	Surgical, % (N)	53.5% (354,583)
**Ventilated**	% (N)	37.8% (250,411)
**Top 5 diagnoses**	Coronary artery bypass grafts	46,510 (7.0%)
	Orthopaedic surgery	28,544 (4.3%)
	GI neoplasm	27,983 (4.2%)
	Valvular heart surgery	26,703 (4.0%)
	Drug overdose	23,043 (3.5%)
**Year of admission, N (%)**	2011	118,686 (17.9%)
	2012	127,563 (19.3%)
	2013	123,034 (19.9%)
	2014	143,271 (21.6%)
	2015	140,971 (21.3%)
**Hospital Type**	Rural/Regional [N = 40] n(%)	88,769 (13.4%)
	Metropolitan [N = 36] n(%)	114,998 (17.4%)
	Tertiary/Teaching [N = 38] n(%)	288,242 (43.5%)
	Private [N = 54] n(%)	170,516 (25.7%)
**ICU Admission source**	Operating Theatre / Recovery	343,617 (51.9%)
	Accident & Emergency	168,667 (25.5%)
	Ward	105,631 (16.0%)
	Other ICU, same hospital	1,507 (0.2%)
	Other hospital	37,428 (5.7%)
	Other hospital ICU	5,333 (0.8%)
**Hospital Admission source**	Home, % (N)	76.3% (505,526)
	Other Acute Hospital, %(N)	17.0% (112,721)
	Chronic Care Hospital, %(N)	0.8% (5,393)
	Other Hospital ICU	0.9% (5,688)
	Unknown	5.0% (33,197)
**Severity**	Mean ANZROD^b^	0.097
	Median ANZROD (IQR)	0.022 (0.007–0.089)
	Mean APACHEIII Risk of Death	0.131
	Median APACHEIII Risk of Death (IQR)	0.043 (0.013–0.148)
	Hospital Mortality, %(N)	60,859 (9.2%)

^a^ Percentages refer to proportion of non-missing values

^b^ Australian and New Zealand Risk of Death Score (ANZROD)

### LOS prediction model

The final model is detailed in the supplementary material (S1 and S2 Tables). Those patients admitted with treatment limitation orders, for palliative care, or for organ donation had a shorter LOS than those admitted for full active management. Patients ventilated in the first 24 hours had a longer duration of stay than those patients admitted without ventilation. ANZROD decile was positively associated with LOS and mortality was associated with a decreased LOS. The coefficients and final groupings for each of the 50 diagnoses considered for inclusions are detailed in the supplementary material.

### Model validation

Using only the fixed effects, the coefficient of determination was 0.25 at the individual level suggesting that 25% of the variation in LOS was explained by patient-level factors in the model. Mean observed LOS was highest in Tertiary ICUs, lowest in Private ICUs, and non-elective patients on average stayed longer than elective patients (Table 2). There was no significant difference in the observed and predicted values by hospital classification or elective status (Table 2).

**Table 2:** Observed mean LOS, observed and predicted geometric LOS, p value for difference in observed and predicted ln(LOS) by hospital-type and elective status.

	Length of Stay		Geometric Mean (95% CI)		p
	Mean	Median (IQR)	Observed	Predicted	
*Hospital Classification*					
Rural/Regional	3.08	1.85 (0.92–3.67)	1.81 (1.80 to 1.82)	1.80 (1.79 to 1.81)	0.1545
Metropolitan	3.45	1.96 (0.97–3.94)	2.03 (2.01 to 2.04)	2.02 (2.02 to 2.03)	0.7962
Tertiary	3.67	1.89 (0.95–3.92)	2.06 (2.05 to 2.07)	2.06 (2.06 to 2.07)	0.5979
Private	2.29	1.18 (0.86–2.33)	1.53 (1.52 to 1.53)	1.52 (1.52 to 1.53)	0.1320
*Elective Status*					
Elective	2.20	1.11 (0.87–2.17)	1.48 (1.48 to 1.49)	1.48 (1.48 to 1.49)	0.9553
Non-Elective	3.96	2.16 (1.05–4.49)	2.23 (2.22 to 2.24)	2.23 (2.22 to 2.23)	0.1760

**Fig 1:**
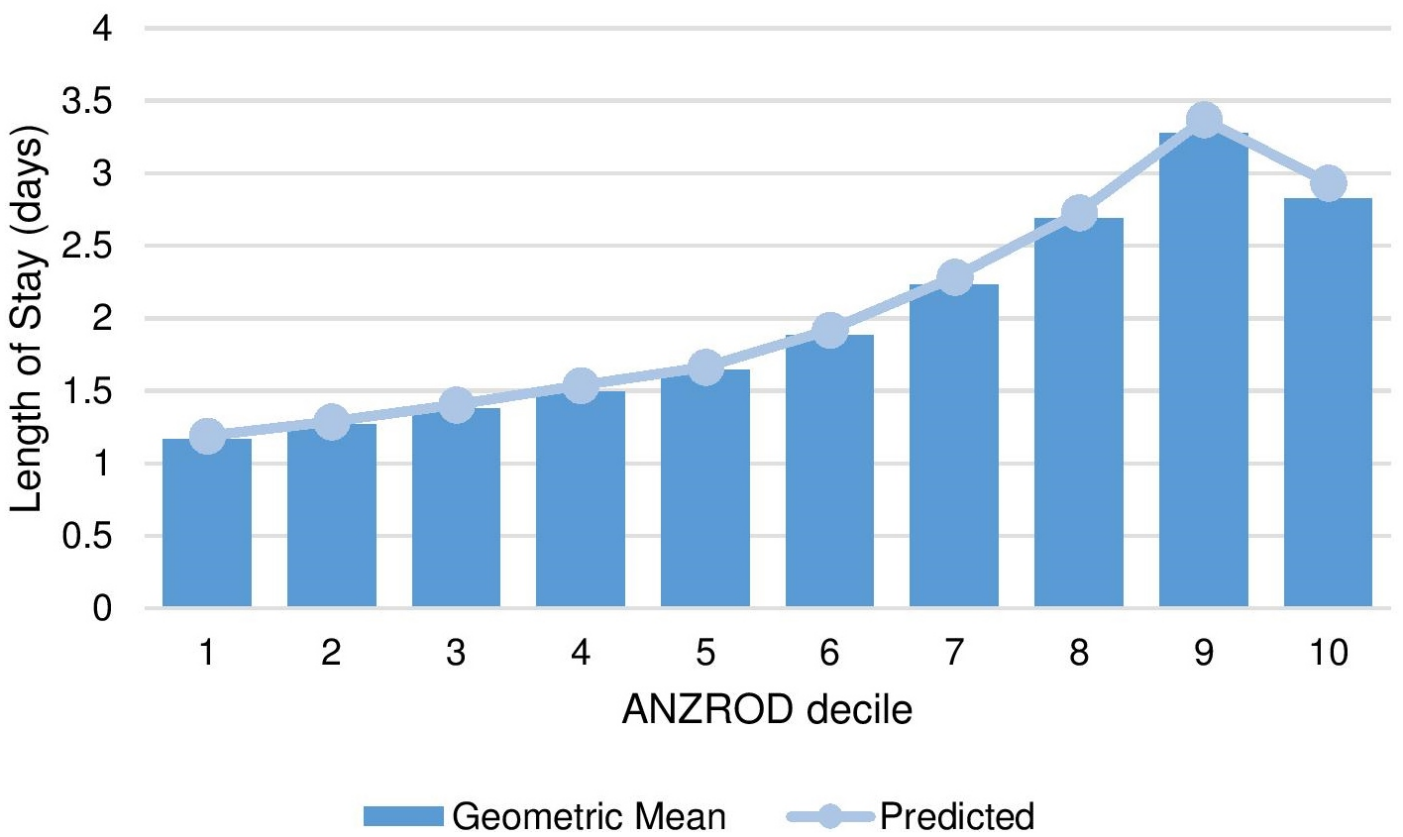
Observed and expected geometric mean LOS by ANZROD decile.

There was strong concordance between the observed and expected geometric mean LOS by ANZROD decile (Fig 1) and age group (S1 Fig), with concordance correlation coefficients of 0.998 and 0.903 respectively.

Among all units, the coefficient of determination between RALOSR and mean LOS was 0.38. When we restricted the units to those between the 25^th^ and 75^th^ percentiles of mean LOS, the coefficient of determination was 0.01 suggesting good risk-adjustment for case-mix among these units.

The linear mixed effects model to evaluate consistency of measures over time demonstrated very strong agreement between the fitted value and the RALOSR. The coefficient of determination was 0.91, suggesting that the estimates were not fluctuating year by year and that unit differences accounted for the majority of unmeasured variation.

### Sensitivity and uncertainty

The accuracy of the estimate of RALOSR for a site using different numbers of observations is detailed in the supplementary material (S3 Table). Accuracy was poor with only 5 observations, with only 23.0% of RALOSR estimates within 10% of the estimate calculated using all observations. At 1000 observations, all but one site-year estimate was within 5% of the final estimate, and all were within 10%.

**Fig 2:**
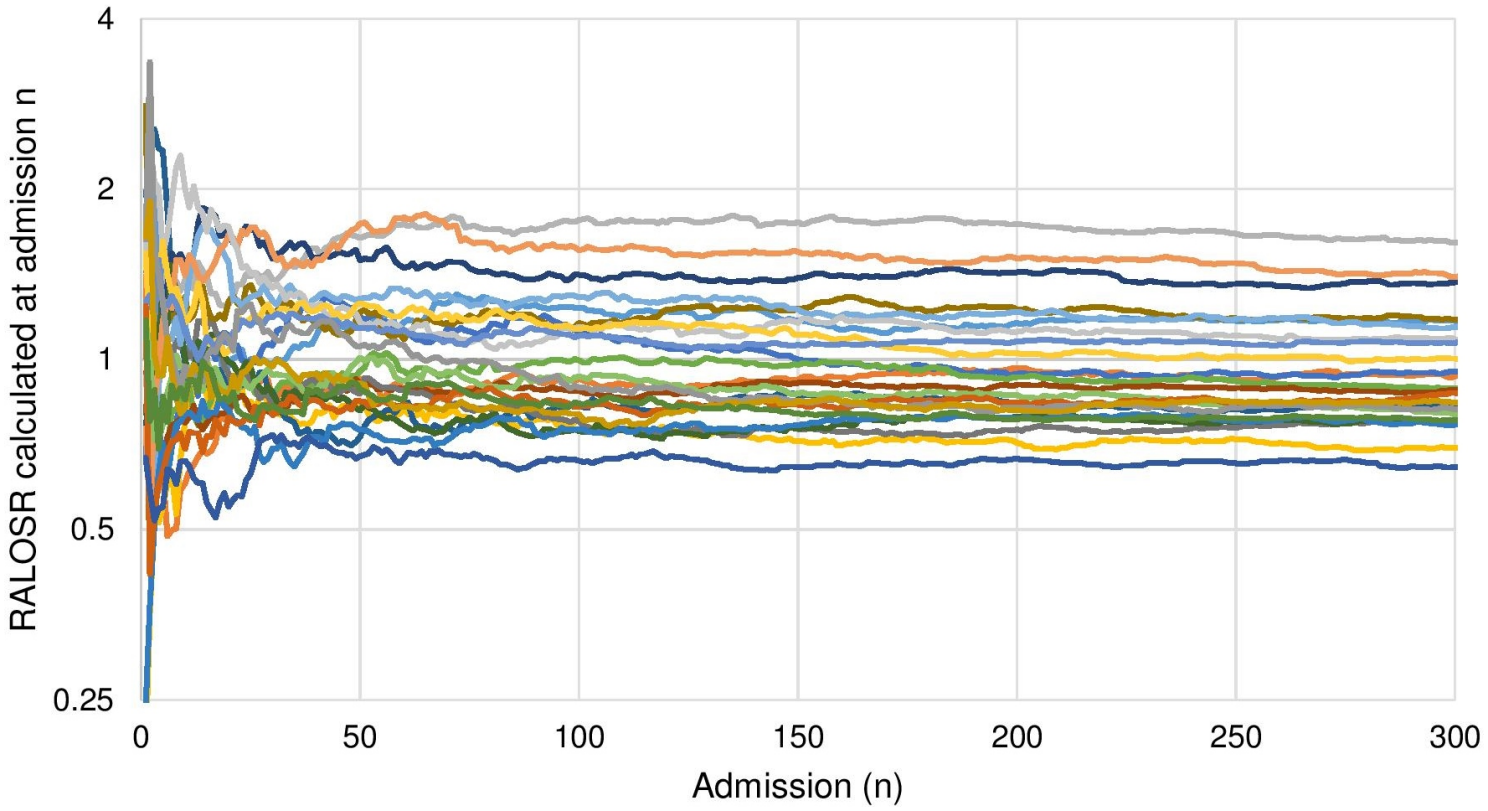
Estimates of the RALOSR for 2015 sites with >1500 admissions by number of admissions used in estimate.

Fig 2 shows the estimate of the RALOSR derived from n admissions by site using 2015 data, which for clarity presents only those sites with over 1500 admissions. Estimates vary significantly when calculated using a low number of admission numbers but converge on a stable estimate at around 200 observations.

Detailed methods and estimates of the uncertainty estimates are provided as supplementary material (S1 File). The uncertainty interval function is given in S2 Fig. When observations were few, uncertainty intervals were wide. Using these intervals the rate of misclassification was very low (S4 Table).

**Fig 3:**
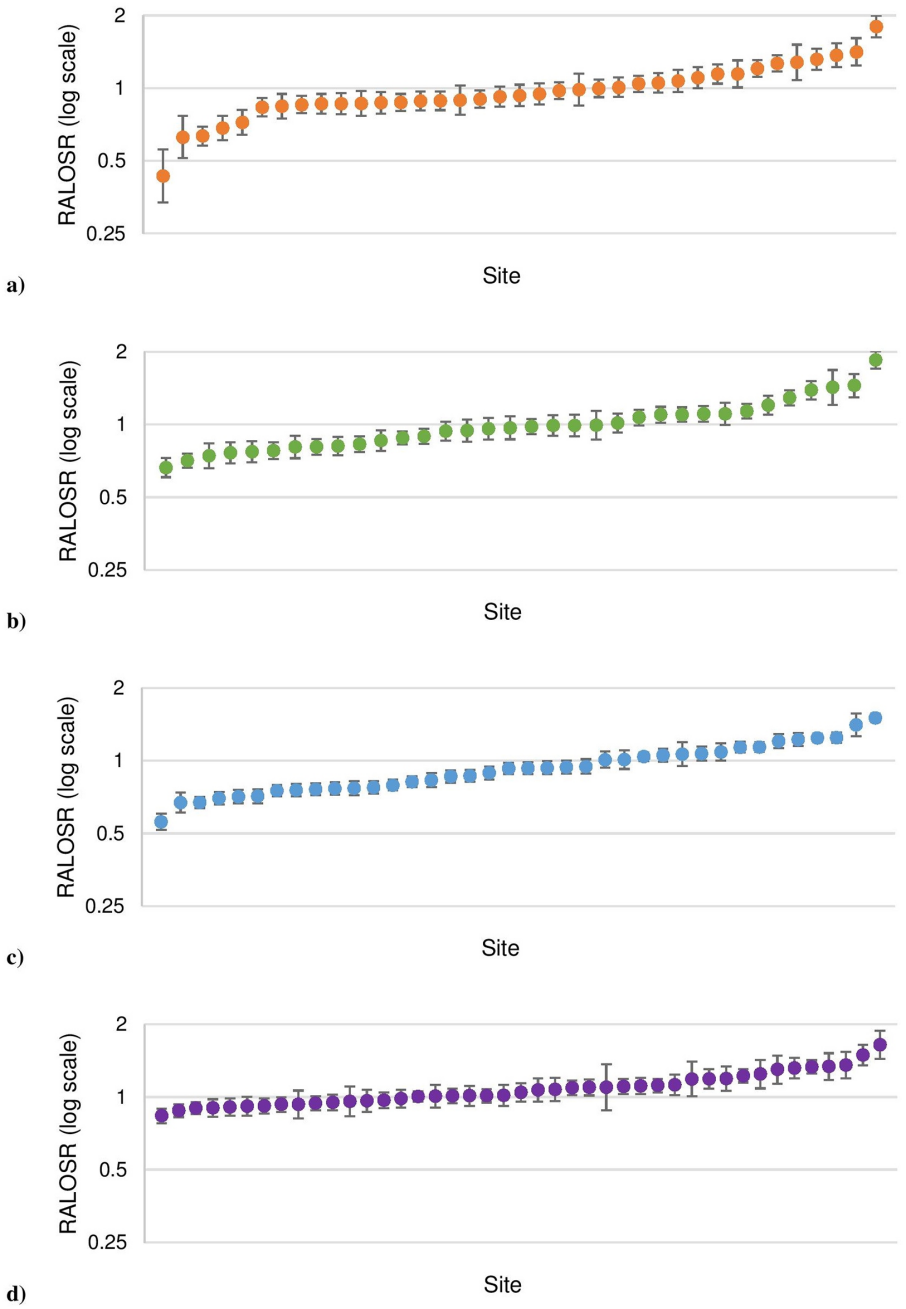
Risk-adjusted LOS ratio by site for 2015, a) Rural/Regional, b) Metropolitan, c) Teaching/Tertiary, and d) Private.

Individual site results for 2015 are given in Fig 3. There were large disparities in the RALOSR with individual site estimates ranging from 0.35 (95% UI: 0.23–0.52) to 2.34 (95% UI: 2.05–2.66). This suggests that there are significant differences in LOS among sites, even after adjusting for patient case-mix.

## Discussion

We describe a multivariable model which can be used to predict length of stay in ICU using routinely collected admission and patient-level data. Comparing predicted with observed LOS permits the calculation of an institutional risk-adjusted metric, the RALOSR which facilitates identification of units which have longer or shorter risk-adjusted length of ICU stay. We found the site-level RALOSR was stable over time, particularly when calculated using more than 200 admissions.

A patient’s ANZROD score was positively associated with increased LOS with the exception of the highest risk decile. We found that this was due to higher mortality in this group and a shorter LOS among patients who died. We demonstrated that this association became linear after accounting for death in the model. Though some studies have excluded patients who died, previous work has shown that there may be significant variability in LOS among ICU non-survivors.[14] We tested the impact of excluding deaths on the LOS association and on the estimated RALOSR (results not shown). As expected, among survivors, the coefficient for the highest ANZROD decile became largest, however we found that their exclusion did not significantly impact our estimates of RALOSR.

Risk-adjusted ICU length of stay has proved challenging to predict accurately.[10, 15] One reason for this may be due to large differences in patient management approaches among ICUs. One Swiss study indicated that the use of discharge guidelines was limited and there was marked heterogeneity in discharge practices among 55 ICUs.[16] Importantly, we have demonstrated that our risk-adjusted estimate (the RALOSR), is consistent for units over time. This suggests that the majority of variation not captured by the model may in fact be attributable to the differences in patient management and discharge practices we seek to measure.

Exploring differences in RALOSR between hospitals may provide an opportunity to reduce health care system costs, through facilitating more efficient ICU utilization. For example, those units with low or moderate RALOSR’s may have systems to more appropriately discharge and manage some patients on the ward. Conversely, units with a high RALOSR may not have appropriate step-down facilities available. It’s likely also that the use of different therapies will be important determinants of LOS. Although, this model controls only for ventilation, identifying high or low RALOSR units will allow us to identify units where LOS is influenced by therapies or other factors such as staffing models. Conceivably, hospital administrators may also be able to use the RALOSR to track the impact of various interventions over time.

Given we use both the ANZROD score and subscores used in its calculation, it’s likely that there are significant collinearities between our predictors. Predictive modelling is more flexible than epidemiological modelling which seeks to understand the relationship between exposures or characteristics and outcomes. In predictive modelling collinearity is less concerning, and typically all variables that improve the predictive performance of the model are included. However as a result, we should be cautious to interpret the associations between predictors and LOS. Of greater concern is overfitting, particularly with a large dataset. To deal with this, we considered the significance of predictors in a 20% subsample of the data and only retained those variables which remained significant.

The extent to which higher or lower LOS is correlated with mortality in this population is not clearly understood. That is, do patients who are managed longer in the ICU have better survival than those who are discharged or transferred to the ward earlier? Previous work in Australian and New Zealand Pediatric ICUs suggests that lower adjusted length of stay is associated with lower rates of mortality.[5] This finding was consistent with a recent US study which showed that increasing LOS was associated with higher 1-year mortality, although this study did not adjust for differences in case-mix for length of stay.[17] It is not clear if such an association exists in our adult population and this remains an important area of future research.

Our study has a number of limitations. First, the possible reasons for reported variation in LOS are diverse; LOS is affected by many intrinsic patient factors. While our model accounts for some of these, there may be variation in patient case-mix not explained by the variables available in our analysis. Second, we use patient characteristics available at the time of admission; thus it is not possible to disentangle the relative contributions to LOS of discharge practice versus the changes in the state of the patient due to deterioration or complications. However, this model will permit us to identify those units with longer LOS than expected and then identify the factors, such as bed block and differences in therapies, which may be driving the disparities. Future studies should concurrently examine clinical outcomes such as mortality, nosocomial infections and complications with RALOSR. Third, our model does not perform well at the individual level and so may have limited application as a prognostic tool at the patient level. Nevertheless, we have demonstrated that the model provided a reliable risk-adjusted metric at the site level. The findings from this study are supported by the use of high quality data with a very large number of admissions. In addition, we found that our findings predicted well in patient subgroups and were robust to a range of modelling assumptions including the exclusion of patients who died.

## Conclusion

We found it was possible to predict ICU LOS for the purposes of identifying site level variation. There were large and statistically significant differences in risk-adjusted LOS among Australian and New Zealand ICUs which persisted over time. This may reflect differences in site level discharge practice. Further work is required to determine the causes of these differences.

## Supporting information

S1 Fig Observed and expected geometric mean LOS by age group. (DOCX)

S2 Fig Standard deviation of errors (ln(RALOSR_N_)–ln(RALOSR_n_)). (DOCX)

S1 File Estimating uncertainty. (DOCX)

S1 Table LOS prediction model for Adult ICU admissions. (DOCX)

S2 Table Diagnostic groupings for weighting in the LOS model. (DOCX)

S3 Table Difference between the risk-adjusted LOS ratio (RALOSR) estimated with various numbers of observations and the final RALOSR estimate using all admissions. (DOCX)

S4 Table Misclassification of units based on RALOSR calculated with various numbers of observations compared with the final RALOSR estimate using all admissions. (DOCX)
