# Supplementary material for: Modelling risk-adjusted variation in length of stay among Australian and New Zealand ICUs
Source: PLoS One. 2017 May 2;12(5):e0176570. doi: 10.1371/journal.pone.0176570 (PMC5413040; doi:10.1371/journal.pone.0176570)
Supplement: S1 Fig — (DOCX) [file pone.0176570.s001.docx]

**S1 Figure. Observed and expected geometric mean LOS by age group**
