# Supplementary material for: Modelling risk-adjusted variation in length of stay among Australian and New Zealand ICUs
Source: PLoS One. 2017 May 2;12(5):e0176570. doi: 10.1371/journal.pone.0176570 (PMC5413040; doi:10.1371/journal.pone.0176570)
Supplement: S1 File — (DOCX) [file pone.0176570.s003.docx]

**S1 File. Estimating Uncertainty**

**Method**

We calculated an uncertainty interval using the standard deviation of the distribution of errors at n=1, 2, 3, 4,…, 500. We calculated the difference between the Ln(RALOSR) calculated with observations=1, 2, 3, 4,…, 500 and the Ln(RALOSR) that would be estimated at the final admission among those site-years with >1000 admission in a year (ΔRALOSR). The standard deviation of the error at each observation value can provide an estimate of the standard error of the RALOSR. We expressed the standard deviation as a function of the number of observations to provide a generalized approach for estimating an uncertainty interval. Given we have a finite number of observations, as the observation number approaches the total admissions, ΔRALOSR will approach 0. For this reason, we only used data to the 500^th^ observation to avoid biasing our errors toward zero.

**Results**

The standard deviation of ΔRALOSR is plotted against the number of admissions used to calculate the RALOSR estimate in S2 Fig. When a small number of admissions are used, the uncertainty is large and decreases rapidly as the number of admissions reaches 50. The generalised equation for the 95% uncertainty interval is given by:

$$RUi\pm0.736\times\left( n^{-0.433} \right)$$

Where n is the number of admissions.
