# Supplementary material for: Modelling risk-adjusted variation in length of stay among Australian and New Zealand ICUs
Source: PLoS One. 2017 May 2;12(5):e0176570. doi: 10.1371/journal.pone.0176570 (PMC5413040; doi:10.1371/journal.pone.0176570)
Supplement: S1 Table — (DOCX) [file pone.0176570.s004.docx]

**S1 Table. LOS prediction model for Adult ICU admissions**

| ***ANZROD Decile*** |  |  |
| --- | --- | --- |
| 1 | *Reference* |  |
| 2 | 0.127 (0.118 to 0.136) | <0.001 |
| 3 | 0.225 (0.216 to 0.235) | <0.001 |
| 4 | 0.318 (0.308 to 0.328) | <0.001 |
| 5 | 0.421 (0.411 to 0.431) | <0.001 |
| 6 | 0.540 (0.529 to 0.550) | <0.001 |
| 7 | 0.677 (0.666 to 0.688) | <0.001 |
| 8 | 0.837 (0.825 to 0.849) | <0.001 |
| 9 | 1.039 (1.026 to 1.052) | <0.001 |
| 10 | 1.068 (1.051 to 1.084) | <0.001 |
| Died in ICU | -0.506 (-0.517 to -0.496) | <0.001 |
| Ventilated | 0.423 (0.417 to 0.429) | <0.001 |
| ***Age Category*** |  |  |
| 18 to <30 years | *Reference* |  |
| 30 to <40 years | 0.049 (0.038 to 0.060) | <0.001 |
| 40 to <50 years | 0.066 (0.055 to 0.076) | <0.001 |
| 50 to <60 years | 0.061 (0.051 to 0.071) | <0.001 |
| 60 to <70 years | -0.018 (-0.028 to -0.008) | <0.001 |
| 70 to <80 years | -0.080 (-0.090 to -0.070) | <0.001 |
| 80 to < 90 years | -0.173 (-0.185 to -0.162) | <0.001 |
| > 90 years | -0.351 (-0.371 to -0.331) | <0.001 |
| ***APACHE III - subscores**** |  |  |
| Albumin Score | 0.011 (0.010 to 0.012) | <0.001 |
| Bilirubin Score | 0.009 (0.008 to 0.010) | <0.001 |
| Creatinine Score | 0.012 (0.011 to 0.013) | <0.001 |
| Haematocrit Score | 0.060 (0.058 to 0.062) | <0.001 |
| Heart Rate Score | 0.014 (0.014 to 0.015) | <0.001 |
| Oxygenation Score | 0.011 (0.010 to 0.011) | <0.001 |
| pH Score | 0.008 (0.007 to 0.009) | <0.001 |
| Temperature Score | -0.003 (-0.004 to -0.002) | <0.001 |
| Urea Score | 0.010 (0.009 to 0.011) | <0.001 |
| Urine Output Score | -0.016 (-0.017 to -0.016) | <0.001 |
| Glasgow Coma Score | 0.012 (0.011 to 0.013) | <0.001 |
| ***ICU Source*** |  |  |
| Operating theatre/recovery | *Reference* |  |
| Emergency department | 0.090 (0.079 to 0.101) | <0.001 |
| Ward | 0.115 (0.104 to 0.126) | <0.001 |
| Other ICU, same hospital | 0.225 (0.181 to 0.268) | <0.001 |
| Other hospital | 0.220 (0.207 to 0.233) | <0.001 |
| Other hospital ICU | 0.424 (0.395 to 0.452) | <0.001 |
| ***Hospital Source*** |  |  |
| Home | *Reference* |  |
| Other Acute Hospital | 0.014 (0.008 to 0.021) | <0.001 |
| Chronic Care Hospital | -0.134 (-0.157 to -0.112) | <0.001 |
| Other Hospital ICU | 0.136 (0.109 to 0.163) | <0.001 |
| Unknown | -0.045 (-0.065 to -0.025) | <0.001 |
| Surgical | 0.096 (0.086 to 0.106) | <0.001 |
| ***Treatment goals*** |  |  |
| Full active management | *Reference* |  |
| Treatment limitation order | -0.313 (-0.324 to -0.301) | <0.001 |
| Palliative Care | -1.021 (-1.058 to -0.983) | <0.001 |
| Organ donation | -0.735 (-0.800 to -0.669) | <0.001 |
| Chronic Cardiovascular Disease | 0.057 (0.049 to 0.065) | <0.001 |
| Chronic Liver Disease | -0.115 (-0.131 to -0.099) | <0.001 |
| Lymphoma | -0.049 (-0.071 to -0.027) | <0.001 |
| Metastases | -0.224 (-0.235 to -0.213) | <0.001 |
| Immunosuppression | -0.041 (-0.052 to -0.030) | <0.001 |
| ***LOS Group based on Diagnosis^a^*** |  |  |
| 1 | 0.630 (0.600 to 0.660) | <0.001 |
| 2 | 0.393 (0.381 to 0.404) | <0.001 |
| 3 | 0.253 (0.245 to 0.261) | <0.001 |
| 4 | 0.165 (0.154 to 0.176) | <0.001 |
| 5 | -0.156 (-0.163 to -0.149) | <0.001 |
| 6 | -0.242 (-0.250 to -0.234) | <0.001 |
| 7 | -0.255 (-0.268 to -0.243) | <0.001 |
| Constant | -0.458 (-0.499 to -0.417) | <0.001 |

^a^Table s2
