# Supplementary material for: Modelling risk-adjusted variation in length of stay among Australian and New Zealand ICUs
Source: PLoS One. 2017 May 2;12(5):e0176570. doi: 10.1371/journal.pone.0176570 (PMC5413040; doi:10.1371/journal.pone.0176570)
Supplement: S2 Table — (DOCX) [file pone.0176570.s005.docx]

**S2 Table. Diagnostic groupings for weighting in the LOS model**

| **Diagnostic Group** | **APD diagnostic code** | **Diagnosis** | **β** |
| --- | --- | --- | --- |
| 1 | 406 | Neuromuscular disease | 0.748 |
| 1 | 210 | Parasitic pneumonia | 0.589 |
| 1 | 604 | Multi trauma with spinal injury | 0.515 |
| 2 | 1601 | Head trauma +/- multi trauma - post operative | 0.496 |
| 2 | 602 | Multiple trauma excluding head | 0.396 |
| 2 | 213 | Viral pneumonia | 0.395 |
| 2 | 605 | Isolated cervical spine injury | 0.389 |
| 2 | 311 | Pancreatitis | 0.349 |
| 2 | 1604 | Multi trauma with spinal injury - post operative | 0.349 |
| 2 | 404 | Neurologic infection | 0.312 |
| 3 | 1503 | Subarachnoid haemorrhage | 0.270 |
| 3 | 212 | Bacterial pneumonia | 0.270 |
| 3 | 1501 | Intracerebral haemorrhage | 0.263 |
| 3 | 402 | Subarachnoid haemorrhage | 0.230 |
| 3 | 1602 | Multiple trauma excluding head - post operative | 0.227 |
| 3 | 702 | Diabetic ketoacidosis | 0.226 |
| 3 | 209 | Asthma | 0.224 |
| 4 | 201 | Aspiration pneumonia | 0.193 |
| 4 | 601 | Head trauma +/- multi trauma | 0.161 |
| 4 | 1407 | Liver transplant | 0.160 |
| 4 | 1212 | CABG with valve repair/replacement | 0.122 |
| *Reference (along with all other diagnoses*) | 1401 | GI perforation/rupture (not peritonitis) | 0.071 |
|  | 503 | Sepsis with shock, other than urinary | 0.050 |
|  | 1207 | Coronary artery bypass grafts | 0.034 |
|  | 1206 | Valvular heart surgery | 0.032 |
|  | 1205 | Carotid endarterectomy | 0.010 |
|  | 2201 | Metabolic disease | -0.019 |
|  | 1408 | Other GI diseases | -0.021 |
|  | 1504 | Laminectomy/Spinal cord surgery | -0.076 |
|  | 1302 | Respiratory neoplasm – lung | -0.077 |
| 5 | 1902 | Orthopaedic surgery | -0.114 |
| 5 | 1903 | Skin surgery | -0.114 |
| 5 | 1506 | Other neurologic disease | -0.128 |
| 5 | 104 | Congestive heart failure | -0.137 |
| 5 | 305 | GI bleeding – ulcer/laceration | -0.155 |
| 5 | 1304 | Other respiratory diseases | -0.158 |
| 5 | 1505 | Craniotomy for neoplasm | -0.183 |
| 5 | 1213 | Endoluminal aortic repair | -0.193 |
| 6 | 1403 | GI bleeding | -0.210 |
| 6 | 1208 | Other cardiovascular diseases - post operative | -0.210 |
| 6 | 109 | Other cardiovascular disease | -0.244 |
| 6 | 1203 | Peripheral artery bypass graft | -0.253 |
| 6 | 1002 | Other medical disorders | -0.267 |
| 6 | 403 | Stroke | -0.272 |
| 6 | 1202 | Peripheral vascular disease | -0.279 |
| 6 | 101 | Cardiogenic shock | -0.287 |
| 6 | 106 | Rhythm disturbance | -0.290 |
| 7 | 102 | Cardiac arrest | -0.307 |
| 7 | 401 | Intracerebral haemorrhage | -0.320 |
| 7 | 107 | Acute myocardial infarction | -0.332 |
