# Supplementary material for: Modelling risk-adjusted variation in length of stay among Australian and New Zealand ICUs
Source: PLoS One. 2017 May 2;12(5):e0176570. doi: 10.1371/journal.pone.0176570 (PMC5413040; doi:10.1371/journal.pone.0176570)
Supplement: S3 Table — (DOCX) [file pone.0176570.s006.docx]

**S3 Table. Difference between the Risk-adjusted LOS ratio (RALOSR) estimated with various numbers of observations and the final RALOSR estimate using all admissions.**

|  | **Absolute difference** | | **Proportion of sites with a difference <x%** | | |
| --- | --- | --- | --- | --- | --- |
| **Observations**  **(n)** | **Mean** | **(Range)** | **x=15%** | **x=10%** | **x=5%** |
| **5** | 0.27 | (0.00 to 1.57) | 36.7% (83) | 23.0% (52) | 12.4% (28) |
| **10** | 0.19 | (0.00 to 1.44) | 49.1% (111) | 31.9% (72) | 14.2% (32) |
| **50** | 0.08 | (0.00 to 0.38) | 82.7% (187) | 63.7% (144) | 38.1% (86) |
| **100** | 0.06 | (0.00 to 0.27) | 92.0% (208) | 77.0% (174) | 42.9% (97) |
| **200** | 0.04 | (0.00 to 0.15) | 99.6% (225) | 92.9% (210) | 63.3% (143) |
| **300** | 0.03 | (0.00 to 0.12) | 100.0% (226) | 98.2% (222) | 75.2% (170) |
| **500** | 0.02 | (0.00 to 0.10) | 100.0% (226) | 100.0% (226) | 91.2% (206) |
| **1000** | 0.01 | (0.00 to 0.08) | 100.0% (226) | 100.0% (226) | 99.6% (225) |
