# Supplementary material for: Modelling risk-adjusted variation in length of stay among Australian and New Zealand ICUs
Source: PLoS One. 2017 May 2;12(5):e0176570. doi: 10.1371/journal.pone.0176570 (PMC5413040; doi:10.1371/journal.pone.0176570)
Supplement: S4 Table — (DOCX) [file pone.0176570.s007.docx]

**S4 Table. Misclassification of units based on RALOSR calculated with various numbers of observations compared with the final RALOSR estimate using all admissions.**

| **Final Classification** | **Shorter LOS than expected (LOW)** | | | **Not significantly Different (NSD)** | | | **Longer than expected (LONG)** | | | **Units** | **Misclassification** |
| --- | --- | --- | --- | --- | --- | --- | --- | --- | --- | --- | --- |
| **Interim Classification** | **LOW** | **NSD** | **LONG** | **LOW** | **NSD** | **LONG** | **LOW** | **NSD** | **LONG** |  |  |
| 5 | 9 | 110 | 1 |  | 43 | 1 |  | 58 | 4 | 226 | 0.9% |
| 10 | 14 | 104 | 2 |  | 43 | 1 |  | 56 | 6 | 226 | 1.3% |
| 50 | 50 | 70 |  | 1 | 42 | 1 |  | 44 | 18 | 226 | 0.9% |
| 100 | 68 | 52 |  | 1 | 42 | 1 |  | 34 | 28 | 226 | 0.9% |
| 200 | 79 | 41 |  |  | 43 | 1 |  | 27 | 35 | 226 | 0.4% |
| 300 | 88 | 32 |  |  | 42 | 2 |  | 15 | 47 | 226 | 0.9% |
| 500 | 99 | 21 |  |  | 43 | 1 |  | 13 | 49 | 226 | 0.4% |
| 1000 | 111 | 9 |  |  | 42 | 2 |  | 4 | 58 | 226 | 0.9% |
